# 2-(3-Nitro­phen­yl)-4-oxo-4-phenyl­butane­nitrile

**DOI:** 10.1107/S1600536811021052

**Published:** 2011-06-11

**Authors:** Jingya Yang, Hongyan Zhou, Zheng Li

**Affiliations:** a Key Laboratory of Eco-Environment-Related Polymer Materials, Ministry of Education, Key Laboratory of Polymer Materials of Gansu Province, College of Chemistry and Chemical Engineering, Northwest Normal University, Lanzhou 730070, People’s Republic of China; b College of Science, Gansu Agricultural University, Lanzhou 730070, People’s Republic of China

## Abstract

The structure of the title compound, C_16_H_12_N_2_O_3_, contains two aromatic rings bridged by a C_3_ chain. The dihedral angle between the rings is 67.6 (1)°. No classical hydrogen bonds are not found in the crystal structure.

## Related literature

For the synthesis of the title compound, see: Yang *et al.* (2009 ▶); Yang, Shen & Chen (2010 ▶). For a related structure, see: Yang, Wu & Chen (2010 ▶). For nitrile-containing pharmaceuticals, see: Fleming *et al.* (2010 ▶).
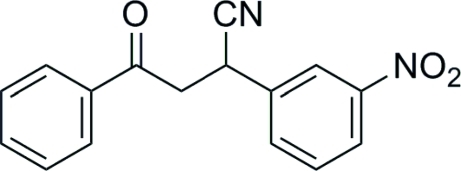


## Experimental

### 

#### Crystal data

C_16_H_12_N_2_O_3_
*M* *_r_* = 280.28
Orthorhombic, 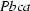

*a* = 10.105 (9) Å
*b* = 8.485 (8) Å
*c* = 31.37 (3) Å
*V* = 2690 (4) Å^3^
*Z* = 8
Mo *K*α radiation
μ = 0.10 mm^−1^
*T* = 296 K
0.28 × 0.25 × 0.24 mm

#### Data collection

Bruker APEXII CCD diffractometer
Absorption correction: multi-scan (*SADABS*; Bruker, 2009 ▶) *T*_min_ = 0.973, *T*_max_ = 0.977
11482 measured reflections
2470 independent reflections
1541 reflections with *I* > 2σ(*I*)
*R*_int_ = 0.064

#### Refinement

*R*[*F* ^2^ > 2σ(*F* ^2^)] = 0.050
*wR*(*F* ^2^) = 0.122
*S* = 1.03
2470 reflections
191 parameters
H-atom parameters constrained
Δρ_max_ = 0.14 e Å^−3^
Δρ_min_ = −0.21 e Å^−3^

### 

Data collection: *APEX2* (Bruker, 2009 ▶); cell refinement: *SAINT* (Bruker, 2009 ▶); data reduction: *SAINT*; program(s) used to solve structure: *SHELXS97* (Sheldrick, 2008 ▶); program(s) used to refine structure: *SHELXL97* (Sheldrick, 2008 ▶); molecular graphics: *SHELXTL* (Sheldrick, 2008 ▶); software used to prepare material for publication: *SHELXTL*.

## Supplementary Material

Crystal structure: contains datablock(s) I, global. DOI: 10.1107/S1600536811021052/bh2356sup1.cif

Structure factors: contains datablock(s) I. DOI: 10.1107/S1600536811021052/bh2356Isup2.hkl

Supplementary material file. DOI: 10.1107/S1600536811021052/bh2356Isup3.cdx

Supplementary material file. DOI: 10.1107/S1600536811021052/bh2356Isup4.cml

Additional supplementary materials:  crystallographic information; 3D view; checkCIF report

## Figures and Tables

**Fig. 1.**
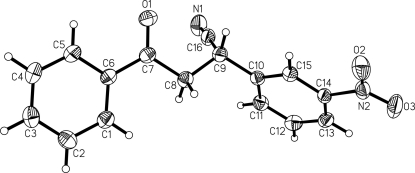
Thermal ellipsoid plot of the title compound at the 30% probability level; hydrogen atoms are drawn as spheres of arbitrary radius.

**Fig. 2.**
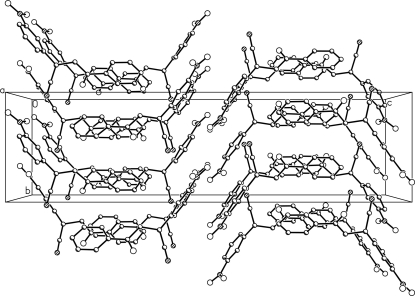
Packing diagram of the title compound; all hydrogen atoms bonded to carbon are omitted for clarity.

## supplementary crystallographic information

### Comment

Nitriles are important synthetic intermediates in organic synthesis, because of their easy preparations and versatile transformations (*e.g*. Yang, Wu & Chen, 2010). Furthermore, nitriles usually exhibit important biological and pharmacological activity. For example, many nitrile-containing pharmaceuticals are widely used in clinical treatments (Fleming *et al.*, 2010).

Here we report on the crystal structure of the title compound, C_16_H_12_N_2_O_3_ (Fig. 1). In the structure, two aromatic rings are planar (the mean deviation from plane of the benzene ring carrying C1 is 0.0025 Å and the corresponding value of the phenyl ring carrying C10 is 0.0043 Å), but they make a large dihedral angle of 67.6 (1)°, which may be caused by the carbon chain of the bridging section that can rotate freely. From the packing diagram (Fig. 2), it can be seen that the molecules of the title compound, which display a similar V-shaped conformation, are interlayed along the *a* axis to form a crab-like motif and these crab-like motifs are repeatedly arranged to generate the final crystal structure.

### Experimental

After Cs_2_CO_3_ (0.5 mg, 0.0015 mmol), (*E*)-3-(3-nitrophenyl)-1-phenylprop-2-en-1-one (76.0 mg, 0.3 mmol), and dioxane (0.5 ml) were charged into a dry Schlenk tube equipped with cold finger under argon, Me_3_SiCN (57 ml, 0.45 mmol) and H_2_O (22 ml, 1.2 mmol) were added. The reaction mixture was refluxed until the reaction was complete (monitored by TLC). Then, H_2_O (2 ml) was added at r.t. and the resulting mixture was extracted with EtOAc (5 ml). The extract was washed with H_2_O (2 ml), brine (3 ml), dried (Na_2_SO_4_), and concentrated. The crude product was purified by flash column chromatography on silica gel (PE–EtOAc, 10:1) to afford pure title compound as a yellowish solid (75.7 mg, 90% yield), as previously reported (Yang *et al.*, 2009; Yang, Shen & Chen, 2010). Colorless single crystals of the title compound suitable for X-ray structure determination were obtained by vapour diffusion of petroleum ether into its ethyl acetate solution at room temperature.

### Refinement

All hydrogen atoms bonded to carbon were introduced to idealized positions and allowed to ride on their parent atoms.

### Crystal data

**Table:**

C_16_H_12_N_2_O_3_	*F*(000) = 1168
*M**_r_* = 280.28	*D*_x_ = 1.384 Mg m^−^^3^
Orthorhombic, *P**b**c**a*	Mo *K*α radiation, λ = 0.71073 Å
Hall symbol: -P 2ac 2ab	Cell parameters from 1926 reflections
*a* = 10.105 (9) Å	θ = 2.6–24.8°
*b* = 8.485 (8) Å	µ = 0.10 mm^−^^1^
*c* = 31.37 (3) Å	*T* = 296 K
*V* = 2690 (4) Å^3^	Block, colourless
*Z* = 8	0.28 × 0.25 × 0.24 mm

### Data collection

**Table:**

Bruker APEXII CCD diffractometer	2470 independent reflections
Radiation source: fine-focus sealed tube	1541 reflections with *I* > 2σ(*I*)
graphite	*R*_int_ = 0.064
φ and ω scans	θ_max_ = 25.5°, θ_min_ = 2.6°
Absorption correction: multi-scan (*SADABS*; Bruker, 2009)	*h* = −11→9
*T*_min_ = 0.973, *T*_max_ = 0.977	*k* = −10→10
11482 measured reflections	*l* = −36→38

### Refinement

**Table:**

Refinement on *F*^2^	Secondary atom site location: difference Fourier map
Least-squares matrix: full	Hydrogen site location: inferred from neighbouring sites
*R*[*F*^2^ > 2σ(*F*^2^)] = 0.050	H-atom parameters constrained
*wR*(*F*^2^) = 0.122	*w* = 1/[σ^2^(*F*_o_^2^) + (0.0432*P*)^2^ + 0.5508*P*] where *P* = (*F*_o_^2^ + 2*F*_c_^2^)/3
*S* = 1.03	(Δ/σ)_max_ < 0.001
2470 reflections	Δρ_max_ = 0.14 e Å^−^^3^
191 parameters	Δρ_min_ = −0.21 e Å^−^^3^
0 restraints	Extinction correction: *SHELXL97* (Sheldrick, 2008), Fc^*^=kFc[1+0.001xFc^2^λ^3^/sin(2θ)]^-1/4^
0 constraints	Extinction coefficient: 0.0057 (9)
Primary atom site location: structure-invariant direct methods	

### Fractional atomic coordinates and isotropic or equivalent isotropic displacement parameters (Å^2^)

**Table:**

	*x*	*y*	*z*	*U*_iso_*/*U*_eq_	
C1	0.9659 (2)	0.7730 (3)	0.76158 (8)	0.0531 (7)	
H1	1.0233	0.8327	0.7449	0.064*	
C2	0.9790 (3)	0.7738 (3)	0.80556 (8)	0.0600 (7)	
H2	1.0458	0.8331	0.8182	0.072*	
C3	0.8944 (3)	0.6881 (3)	0.83050 (8)	0.0611 (8)	
H3	0.9030	0.6898	0.8600	0.073*	
C4	0.7968 (3)	0.5993 (3)	0.81171 (8)	0.0612 (8)	
H4	0.7392	0.5406	0.8286	0.073*	
C5	0.7838 (2)	0.5969 (3)	0.76813 (8)	0.0529 (7)	
H5	0.7177	0.5359	0.7558	0.063*	
C6	0.8680 (2)	0.6842 (2)	0.74222 (7)	0.0431 (6)	
C7	0.8505 (3)	0.6785 (3)	0.69527 (7)	0.0462 (6)	
C8	0.9342 (2)	0.7828 (3)	0.66743 (7)	0.0472 (6)	
H8A	1.0267	0.7642	0.6740	0.057*	
H8B	0.9148	0.8919	0.6741	0.057*	
C9	0.9127 (2)	0.7566 (3)	0.61978 (7)	0.0470 (6)	
H9	0.8180	0.7682	0.6139	0.056*	
C10	0.9863 (2)	0.8761 (2)	0.59265 (6)	0.0411 (6)	
C11	1.1213 (3)	0.8670 (3)	0.58598 (7)	0.0505 (6)	
H11	1.1692	0.7857	0.5985	0.061*	
C12	1.1858 (3)	0.9763 (3)	0.56111 (8)	0.0555 (7)	
H12	1.2767	0.9683	0.5570	0.067*	
C13	1.1164 (3)	1.0971 (3)	0.54237 (7)	0.0512 (7)	
H13	1.1588	1.1705	0.5251	0.061*	
C14	0.9831 (3)	1.1064 (2)	0.54980 (6)	0.0408 (6)	
C15	0.9168 (2)	0.9985 (2)	0.57425 (6)	0.0425 (6)	
H15	0.8260	1.0075	0.5784	0.051*	
C16	0.9514 (3)	0.5952 (3)	0.60812 (7)	0.0543 (7)	
N1	0.9820 (3)	0.4705 (3)	0.60021 (7)	0.0789 (8)	
N2	0.9061 (3)	1.2315 (2)	0.52900 (6)	0.0551 (6)	
O1	0.77009 (19)	0.5898 (2)	0.67920 (5)	0.0685 (6)	
O2	0.7895 (2)	1.2441 (2)	0.53819 (6)	0.0762 (6)	
O3	0.9621 (2)	1.3169 (2)	0.50346 (6)	0.0822 (7)	

### Atomic displacement parameters (Å^2^)

**Table:**

	*U*^11^	*U*^22^	*U*^33^	*U*^12^	*U*^13^	*U*^23^
C1	0.0547 (19)	0.0492 (15)	0.0554 (16)	−0.0029 (13)	0.0082 (13)	−0.0014 (11)
C2	0.063 (2)	0.0625 (17)	0.0547 (16)	0.0048 (15)	−0.0027 (14)	−0.0084 (13)
C3	0.075 (2)	0.0583 (16)	0.0504 (15)	0.0120 (16)	0.0040 (15)	0.0060 (13)
C4	0.068 (2)	0.0549 (16)	0.0612 (17)	0.0003 (15)	0.0160 (15)	0.0127 (13)
C5	0.0537 (18)	0.0455 (14)	0.0595 (16)	−0.0052 (13)	0.0064 (13)	0.0021 (11)
C6	0.0445 (15)	0.0328 (11)	0.0520 (14)	0.0030 (11)	0.0029 (12)	−0.0005 (10)
C7	0.0465 (17)	0.0381 (12)	0.0539 (14)	0.0004 (12)	0.0034 (12)	−0.0021 (11)
C8	0.0552 (17)	0.0396 (13)	0.0469 (13)	−0.0003 (12)	0.0044 (12)	−0.0019 (10)
C9	0.0488 (16)	0.0423 (13)	0.0498 (14)	0.0000 (12)	−0.0024 (12)	0.0033 (11)
C10	0.0458 (16)	0.0380 (13)	0.0396 (12)	0.0008 (12)	−0.0016 (11)	−0.0032 (9)
C11	0.0514 (18)	0.0459 (14)	0.0541 (14)	0.0061 (13)	−0.0062 (13)	−0.0009 (11)
C12	0.0466 (17)	0.0611 (17)	0.0588 (15)	−0.0019 (14)	0.0072 (13)	−0.0091 (13)
C13	0.065 (2)	0.0471 (15)	0.0418 (13)	−0.0128 (14)	0.0069 (13)	−0.0057 (11)
C14	0.0525 (18)	0.0373 (13)	0.0327 (11)	−0.0009 (12)	−0.0002 (11)	−0.0023 (9)
C15	0.0462 (15)	0.0418 (13)	0.0395 (12)	0.0016 (12)	0.0023 (11)	−0.0047 (10)
C16	0.076 (2)	0.0431 (14)	0.0434 (13)	−0.0050 (14)	0.0011 (13)	0.0012 (11)
N1	0.130 (2)	0.0490 (14)	0.0580 (13)	0.0020 (15)	0.0080 (14)	−0.0041 (11)
N2	0.0806 (19)	0.0456 (13)	0.0390 (11)	0.0003 (13)	−0.0050 (12)	0.0016 (9)
O1	0.0680 (14)	0.0775 (13)	0.0598 (11)	−0.0274 (11)	0.0011 (10)	−0.0029 (10)
O2	0.0735 (16)	0.0810 (14)	0.0740 (13)	0.0222 (12)	−0.0015 (12)	0.0186 (10)
O3	0.1196 (19)	0.0640 (12)	0.0631 (12)	−0.0041 (12)	0.0080 (12)	0.0230 (10)

### Geometric parameters (Å, °)

**Table:**

C1—C6	1.384 (3)	C9—C16	1.470 (4)
C1—C2	1.386 (4)	C9—C10	1.518 (3)
C1—H1	0.9300	C9—H9	0.9800
C2—C3	1.368 (4)	C10—C15	1.380 (3)
C2—H2	0.9300	C10—C11	1.381 (3)
C3—C4	1.374 (4)	C11—C12	1.376 (3)
C3—H3	0.9300	C11—H11	0.9300
C4—C5	1.374 (4)	C12—C13	1.374 (3)
C4—H4	0.9300	C12—H12	0.9300
C5—C6	1.390 (3)	C13—C14	1.369 (4)
C5—H5	0.9300	C13—H13	0.9300
C6—C7	1.484 (3)	C14—C15	1.370 (3)
C7—O1	1.217 (3)	C14—N2	1.469 (3)
C7—C8	1.504 (3)	C15—H15	0.9300
C8—C9	1.527 (3)	C16—N1	1.130 (3)
C8—H8A	0.9700	N2—O2	1.218 (3)
C8—H8B	0.9700	N2—O3	1.219 (3)
			
C6—C1—C2	120.5 (2)	C16—C9—C8	109.97 (19)
C6—C1—H1	119.7	C10—C9—C8	112.46 (19)
C2—C1—H1	119.7	C16—C9—H9	107.9
C3—C2—C1	120.4 (3)	C10—C9—H9	107.9
C3—C2—H2	119.8	C8—C9—H9	107.9
C1—C2—H2	119.8	C15—C10—C11	118.8 (2)
C2—C3—C4	119.7 (3)	C15—C10—C9	119.2 (2)
C2—C3—H3	120.2	C11—C10—C9	122.1 (2)
C4—C3—H3	120.2	C12—C11—C10	121.0 (2)
C3—C4—C5	120.2 (3)	C12—C11—H11	119.5
C3—C4—H4	119.9	C10—C11—H11	119.5
C5—C4—H4	119.9	C13—C12—C11	120.3 (3)
C4—C5—C6	121.1 (2)	C13—C12—H12	119.9
C4—C5—H5	119.5	C11—C12—H12	119.9
C6—C5—H5	119.5	C14—C13—C12	118.2 (2)
C1—C6—C5	118.1 (2)	C14—C13—H13	120.9
C1—C6—C7	122.6 (2)	C12—C13—H13	120.9
C5—C6—C7	119.3 (2)	C13—C14—C15	122.5 (2)
O1—C7—C6	120.7 (2)	C13—C14—N2	119.1 (2)
O1—C7—C8	119.9 (2)	C15—C14—N2	118.2 (2)
C6—C7—C8	119.3 (2)	C14—C15—C10	119.2 (2)
C7—C8—C9	113.8 (2)	C14—C15—H15	120.4
C7—C8—H8A	108.8	C10—C15—H15	120.4
C9—C8—H8A	108.8	N1—C16—C9	178.2 (3)
C7—C8—H8B	108.8	O2—N2—O3	123.5 (2)
C9—C8—H8B	108.8	O2—N2—C14	118.1 (2)
H8A—C8—H8B	107.7	O3—N2—C14	118.4 (3)
C16—C9—C10	110.6 (2)		
			
C6—C1—C2—C3	−0.7 (4)	C8—C9—C10—C15	−103.3 (2)
C1—C2—C3—C4	0.7 (4)	C16—C9—C10—C11	−47.2 (3)
C2—C3—C4—C5	−0.2 (4)	C8—C9—C10—C11	76.2 (3)
C3—C4—C5—C6	−0.4 (4)	C15—C10—C11—C12	−0.7 (3)
C2—C1—C6—C5	0.2 (3)	C9—C10—C11—C12	179.7 (2)
C2—C1—C6—C7	−179.5 (2)	C10—C11—C12—C13	0.0 (4)
C4—C5—C6—C1	0.4 (4)	C11—C12—C13—C14	1.1 (3)
C4—C5—C6—C7	−179.9 (2)	C12—C13—C14—C15	−1.5 (3)
C1—C6—C7—O1	174.3 (2)	C12—C13—C14—N2	−178.05 (19)
C5—C6—C7—O1	−5.4 (3)	C13—C14—C15—C10	0.7 (3)
C1—C6—C7—C8	−5.2 (3)	N2—C14—C15—C10	177.34 (18)
C5—C6—C7—C8	175.1 (2)	C11—C10—C15—C14	0.4 (3)
O1—C7—C8—C9	−3.5 (3)	C9—C10—C15—C14	179.96 (19)
C6—C7—C8—C9	176.0 (2)	C13—C14—N2—O2	−175.3 (2)
C7—C8—C9—C16	−62.9 (3)	C15—C14—N2—O2	8.0 (3)
C7—C8—C9—C10	173.33 (19)	C13—C14—N2—O3	4.9 (3)
C16—C9—C10—C15	133.2 (2)	C15—C14—N2—O3	−171.9 (2)
